# Regenerative Therapies to Restore Interneuron Disturbances in Experimental Models of Encephalopathy of Prematurity

**DOI:** 10.3390/ijms22010211

**Published:** 2020-12-28

**Authors:** Josine E. G. Vaes, Chantal M. Kosmeijer, Marthe Kaal, Rik van Vliet, Myrna J. V. Brandt, Manon J. N. L. Benders, Cora H. Nijboer

**Affiliations:** 1 Department for Developmental Origins of Disease, University Medical Center Utrecht Brain Center and Wilhelmina Children’s Hospital, Utrecht University, 3584 Utrecht, The Netherlands; j.e.g.vaes@umcutrecht.nl (J.E.G.V.); C.M.Kosmeijer-3@umcutrecht.nl (C.M.K.); m.kaal@students.uu.nl (M.K.); rik.vanvliet@hotmail.com (R.v.V.); M.J.V.Brandt-3@umcutrecht.nl (M.J.V.B.); 2 Department of Neonatology, University Medical Center Utrecht Brain Center and Wilhelmina Children’s Hospital, Utrecht University, 3584 Utrecht, The Netherlands; m.benders@umcutrecht.nl

**Keywords:** interneurons, encephalopathy of prematurity, neurodevelopmental disorders, regenerative medicine, mesenchymal stem cells, insulin-like growth factor I, preterm birth

## Abstract

Encephalopathy of Prematurity (EoP) is a major cause of morbidity in (extreme) preterm neonates. Though the majority of EoP research has focused on failure of oligodendrocyte maturation as an underlying pathophysiological mechanism, recent pioneer work has identified developmental disturbances in inhibitory interneurons to contribute to EoP. Here we investigated interneuron abnormalities in two experimental models of EoP and explored the potential of two promising treatment strategies, namely intranasal mesenchymal stem cells (MSCs) or insulin-like growth factor I (IGF1), to restore interneuron development. In rats, fetal inflammation and postnatal hypoxia led to a transient increase in total cortical interneuron numbers, with a layer-specific deficit in parvalbumin (PV)+ interneurons. Additionally, a transient excess of total cortical cell density was observed, including excitatory neuron numbers. In the hippocampal cornu ammonis (CA) 1 region, long-term deficits in total interneuron numbers and PV+ subtype were observed. In mice subjected to postnatal hypoxia/ischemia and systemic inflammation, total numbers of cortical interneurons remained unaffected; however, subtype analysis revealed a global, transient reduction in PV+ cells and a long-lasting layer-specific increase in vasoactive intestinal polypeptide (VIP)+ cells. In the dentate gyrus, a long-lasting deficit of somatostatin (SST)+ cells was observed. Both intranasal MSC and IGF1 therapy restored the majority of interneuron abnormalities in EoP mice. In line with the histological findings, EoP mice displayed impaired social behavior, which was partly restored by the therapies. In conclusion, induction of experimental EoP is associated with model-specific disturbances in interneuron development. In addition, intranasal MSCs and IGF1 are promising therapeutic strategies to aid interneuron development after EoP.

## 1. Introduction

Preterm birth is a major cause of neonatal brain injury, leading to significant neurodevelopmental morbidity [1,2,3,4]. In recent years, preclinical and clinical imaging studies have identified distinct patterns of white and (subtle) gray matter deficits in preterm-born neonates, collectively known as Encephalopathy of Prematurity (EoP). These deficits are believed to originate from impaired brain development with peri- and postnatal insults interfering with a multitude of developmental processes that occur in the third trimester of pregnancy [5,6,7].

So far, the majority of research in the EoP field has focused on cerebral white matter injury (WMI), an evident finding by neonatal neuroimaging [8,9,10,11]. Preterm WMI is mainly characterized by widespread (diffuse) hypomyelination, which is the result of maturation failure of the oligodendrocyte lineage [6,12]. However, novel insights in the neurodevelopmental events that take place in the third trimester, combined with improvements in neuroimaging, have led to increased recognition of subtle gray matter deficits contributing to EoP [7,13,14]. GABAergic interneurons have been shown to be develop throughout the third trimester of human gestation, continuing for several months after birth [13,15,16]. Disturbances in the number and function of interneurons have been frequently proposed in the underlying pathophysiology of neurodevelopmental disorders, such as attention deficit disorder (ADD), autism spectrum disorder (ASD), and mood disorders. Interestingly these are neurodevelopmental disorders highly prevalent in the preterm population [14,17,18]. Thus, impairments in interneuron development could play a significant role in EoP pathophysiology and associated neurodevelopmental outcome.

Due to their critical role in the postnatal development of (cortical) circuits and strong link with neurodevelopmental disorders, pioneer studies have explored the vulnerability of interneurons following preterm birth. Recent findings in human post-mortem tissue have revealed cortical interneuron deficits after preterm birth [19,20,21,22]. Aberrations in interneuron development was confirmed in a few, very recent experimental studies, though a large variety of changes in cortical interneuron density and distribution after different preterm-birth related hits were observed [19,21,23,24,25,26,27]. Apart from the limited amount of preclinical evidence on the role of interneurons in EoP pathophysiology, studies exploring potential therapeutic interventions to restore interneuron deficits induced by EoP are currently lacking. Cell- and growth factor-based therapies have received an increasing amount of attention in the field of neonatal brain injury, including that of preterm white matter injury [28,29,30]. One of these emerging treatment options is (intranasal) mesenchymal stem cell (MSC) therapy [28,31]. Administration of MSCs has been shown to boost repair of gray and white matter deficits, dampen neuroinflammation, and improve behavioral outcome in models of term neonatal hypoxia-ischemia and preterm white matter injury [32,33,34,35]. MSCs are believed to exert their regenerative properties through secretion of trophic and anti-inflammatory factors, providing a cerebral milieu permissive for repair and development [28]. Insulin-like growth factor 1 (IGF1) is a trophic factor of which levels are often very low in preterm infants compared to fetal in utero levels at a corresponding gestational age. IGF1 has been demonstrated to play an essential role in normal brain development, including neurogenesis, neuronal differentiation, and neuronal survival [36,37,38,39,40,41]. Moreover, IGF1 treatment was shown to repair white matter injury in models of EoP, boosting maturation and survival of oligodendrocytes [42,43,44,45].

In the present study, we aimed to assess subtype-specific changes in the numbers and distribution of cortical and hippocampal interneurons in two double-hit rodent models of EoP, in which two clinically relevant hits, i.e., fetal inflammation plus postnatal hypoxia or postnatal hypoxia/ischemia plus systemic inflammation, were combined. Moreover, we are the first to explore the potential of two promising treatment strategies in the field of neonatal brain injury, i.e., intranasal MSCs and IGF1, to restore interneuron deficits and improve sociability in our EoP mouse model.

## 2. Results

### 2.1. Fetal Inflammation and Postnatal Hypoxia in Rats Leads to Transient Disturbances in GABAergic Interneuron Distribution in the Cortex and Hippocampus

**Figure 1:**
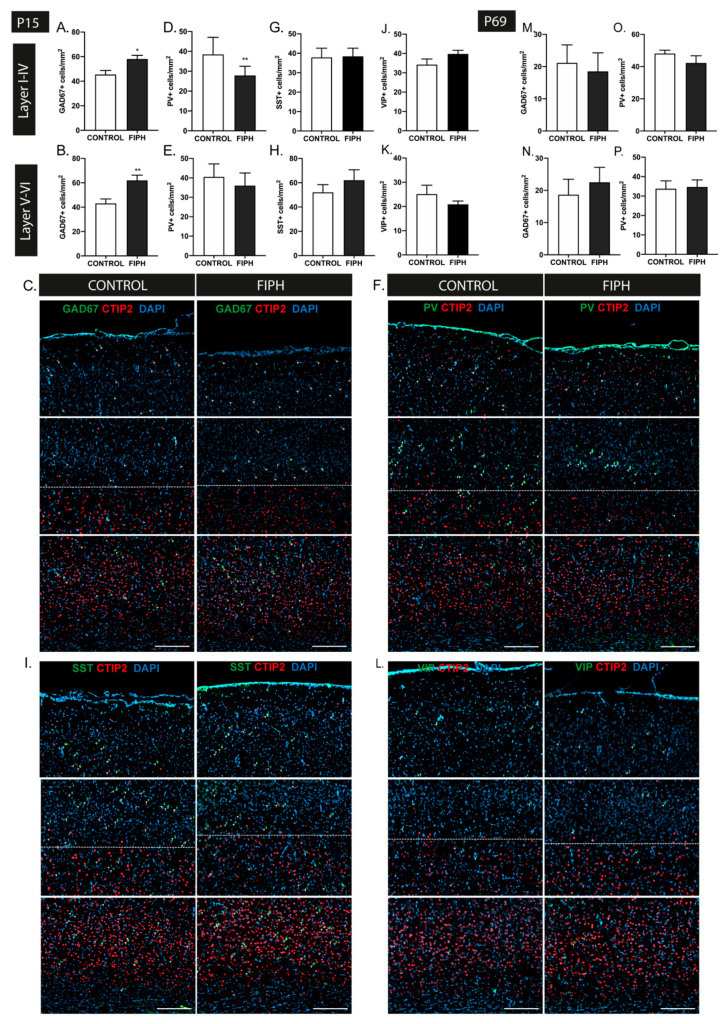
The combination of fetal inflammation and postnatal hypoxia leads to transient disturbances in cortical interneuron density in rats. (**A**,**B**) Animals exposed to the double-hit model displayed an increase in GAD67+ interneuron density in both the upper (**A**) and lower (**B**) cortical layers at P15 (CONTROL *n* = 11, fetal inflammation and postnatal hypoxia (FIPH) *n* = 10). (**C**) Representative fluorescent images of the P15 cortex of a control rat (left) and FIPH rat (right) stained for the general interneuron marker GAD67 (green) and cortical layer marker CTIP2 (red), counterstained with DAPI (blue). GAD67+ cells are indicated with an arrowhead. Dashed line represents the border between upper (I-IV) and lower (V-VI) cortical layers based on CTIP2 expression. Scale bars: 200 µm. (**D**,**E**) A significant decrease in PV+ interneurons per mm^2^ was observed in the upper (**D**) but not the lower (**E**) cortical layers in FIPH rats (CONTROL *n* = 13, FIPH *n* = 8). (**F**) Representative fluorescent images of the P15 cortex of a control (left) and FIPH animal (right) stained for the interneuron subtype marker PV (green) and cortical layer marker CTIP2 (red), counterstained with DAPI (blue). PV+ cells are indicated with an arrowhead. Dashed line represents the border between upper (I-IV) and lower (V-VI) cortical layers. Scale bars: 200 µm. (**G**,**H**) Induction of FIPH did not alter somatostatin (SST)+ cell density at P15 in the upper (**G**) or lower (**H**) cortical layers (CONTROL *n* = 9, FIPH *n* = 9). (**I**) Representative fluorescent images of the P15 cortex of control (left) and FIPH animal (right) stained for the interneuron subtype marker SST (green) and cortical layer marker CTIP2 (red), counterstained with DAPI (blue). SST+ cells are indicated with an arrowhead. Dashed line represents the border between upper (I-IV) and lower (V-VI) cortical layers. Scale bars: 200 µm. (**J**,**K**) The number of vasoactive intestinal polypeptide (VIP)+ interneurons per mm^2^ was not affected in the upper (**J**) or lower (**K**) cortex in our double-hit rat model of Encephalopathy of Prematurity (EoP) (CONTROL *n* = 9, FIPH *n* = 8). (**L**) Representative fluorescent images of the P15 cortex of control (left) and FIPH animal (right) stained for the interneuron subtype marker VIP (green) and cortical layer marker CTIP2 (red), counterstained with DAPI (blue). VIP+ cells are indicated with an arrowhead. Dashed line represents the border between upper (I-IV) and lower (V-VI) cortical layers. Scale bars: 200 µm. (**M**,**N**) At P69, the number of GAD67+ interneurons per mm^2^ endogenously recovered in both the upper (**M**) and lower (**N**) cortical regions of FIPH animals (CONTROL *n* = 6, FIPH *n* = 6). (**O**,**P**) Quantification of PV+ interneurons at P69 revealed endogenous restoration of cell density in the upper cortex (**O**) in FIPH animals, while parvalbumin (PV)+ cell density in the lower (**P**) cortical layers remained unaffected (CONTROL *n* = 9, FIPH *n* = 10). The same group of animals were used for all stainings at P15 and similarly at P69. Animal numbers differ as images with large artefacts or excessive background staining were excluded. *: *p* < 0.05; **: *p* < 0.01 control vs. FIPH animals at the specified time point.

To study the total number of GABAergic interneurons in rats subjected to fetal inflammation and postnatal hypoxia (FIPH), brains sections were stained for the general interneuron marker GAD67 (glutamate decarboxylase), an enzyme essential for GABA synthesis. CTIP2, a protein primarily expressed by neurons localized in layers V-VI, was used to discriminate between upper (I-VI) and lower (V-VI) cortical layers [46]. At P15, a significant increase in the number of interneurons per mm^2^ in both the upper (I-IV) and lower (V-VI) cortical layers of both hemispheres was observed in FIPH rats compared to controls (*p* = 0.014 and *p* = 0.003, respectively) (Figure 1A–C). To gain more insight in interneuron subtype-specific changes, markers for parvalbumin (PV), somatostatin (SST), and vasoactive intestinal polypeptide (VIP) were used. These markers reportedly represent distinct subpopulations of interneurons, covering ~85% of all cortical interneurons [47,48,49]. At P15, a reduction in PV+ interneuron density in the upper cortical layers was observed in FIPH animals compared to controls (*p* = 0.005), but not in the lower cortical layers (*p* = 0.142) (Figure 1D–F). The number of SST+ and VIP+ interneurons per mm^2^ was not affected by FIPH with no differences in either upper or lower cortical layers at P15 in FIPH rats compared to controls (SST: *p* = 0.931 and *p* = 0.363 VIP: *p* = 0.131 and *p* = 0.324 respectively) (Figure 1G–L). In line with our previous data on transient myelination deficits in this FIPH model [50], the observed (layer-specific) changes in GAD67+ and PV+ interneuron density were restored in adulthood (P69) (GAD67: I-IV *p* = 0.746 and V-VI *p* = 0.579 PV: I-IV *p* = 0.254 and V-VI *p* = 0.867; FIPH compared to control rats) (Figure 1M–P).

**Figure 2:**
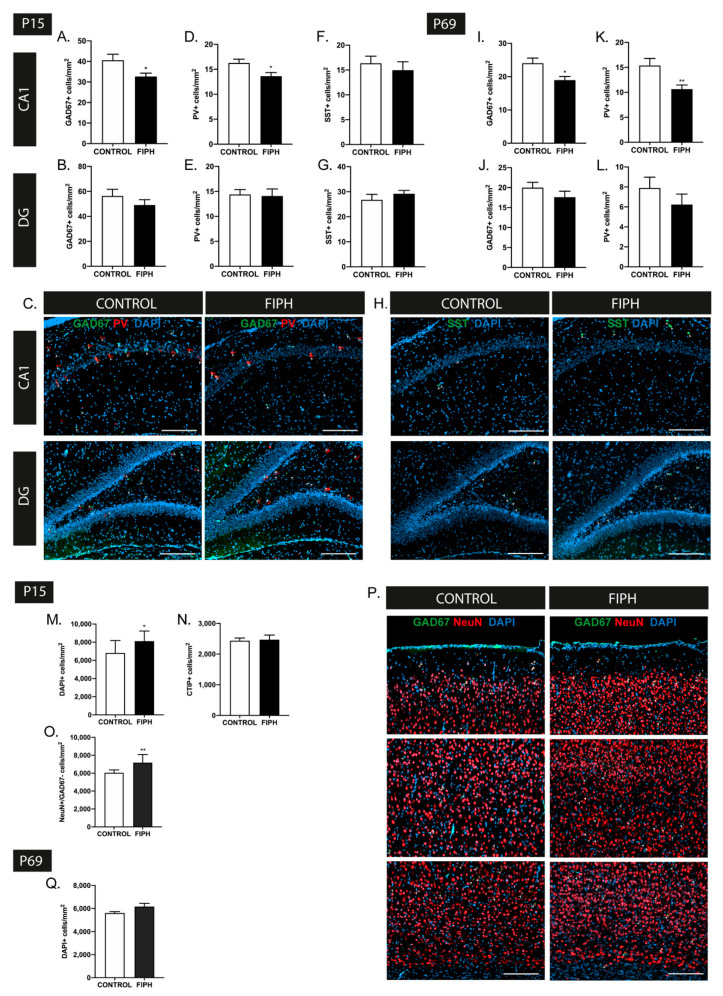
Encephalopathy of Prematurity (EoP) induction is associated with region-specific, persistent changes in hippocampal interneuron density and disruption of cortical development in rats. (**A**,**B**) A significant decrease in GAD67+ cell density was observed in the CA1 region (**A**) of the hippocampus of FIPH animals (*n* = 8) compared to controls (*n* = 7) at P15, while GAD67+ cell density in the dentate gyrus (**B**) was not affected. (**C**) Representative fluorescent images of the CA1 region (upper) and dentate gyrus (lower) of the hippocampus of control (left) and FIPH rats (right), stained with interneuron markers GAD67 (green) and PV (red), counterstained with DAPI (blue). GAD67+ cells are indicated with a white arrowhead and PV+ cells are indicated by a red arrowhead. Scale bars: 200 µm. (**D**,**E**) FIPH animals demonstrate a reduction in PV+ interneurons in the CA1 region (**D**), but not the dentate gyrus (**E**) of the hippocampus at P15 (CONTROL *n* = 8, FIPH *n* = 7). (**F**,**G**) Induction of FIPH did not impact SST+ interneuron density in the CA1 (**F**) region or dentate gyrus (**G**) of the hippocampus at P15 (CONTROL *n* = 7, FIPH *n* = 8). (**H**) Representative fluorescent images of the CA1 region (upper) and dentate gyrus (lower) of the hippocampus of control (left) and FIPH rats (right) stained with interneuron marker SST (green) and counterstained with DAPI (blue). SST+ cells are indicated with a white arrowhead. Scale bars: 200 µm. (**I**,**J**) A reduction in GAD67 cell density was still observed in the CA1 region (**I**) of adult FIPH rats (P69) while the dentate gyrus (**J**) remained unaffected (CONTROL *n* = 9, FIPH *n* = 10). (**K**,**L**) Similar to the findings at P15, at P69 FIPH animals showed a reduced amount of PV+ cells per mm^2^ in the CA1 region (**K**), but not the dentate gyrus (**L**) of the hippocampus (CONTROL *n* = 9, FIPH *n* = 10). (**M**) An increase in DAPI+ cell density was observed in the cortex of FIPH animals at P15 (CONTROL *n* = 13, FIPH *n* = 10). (**N**) No changes in CTIP2+ cell density were observed in the cortex of FIPH animals (*n* = 11), compared to control animals (*n* = 14). (**O**) A higher quantity of NeuN+GAD67- cells was observed in the cortex of FIPH rats (*n* = 6) compared to controls (*n* = 8) at P15, indicative of an excess in excitatory neurons. (**P**) Representative fluorescent images of the cortex of control (left) and FIPH (right) rat, stained for neuronal marker NeuN (red) and interneuron marker GAD67 (green) counterstained with DAPI (blue). White arrowheads indicate double-positive cells. Scale bars: 200 µm. (**Q**) The observed excess in cortical cell density was restored at P69, with no differences in cortical DAPI+ cell numbers between FIPH or control animals (CONTROL *n* = 10, FIPH *n* = 9). The same group of animals were used for all stainings at P15 and similarly at P69. Animal numbers differ as images with large artefacts or excessive background staining were excluded. *: *p* < 0.05; **: *p* < 0.01; control vs. FIPH animals at the specified time point.

The influence of FIPH on hippocampal interneuron density was studied in the CA1 and dentate gyrus (DG) regions as, in line with previous studies [51], we observed the majority of interneurons to reside in these regions. A reduction in GAD67+ and PV+ interneuron density was observed in the CA1 region in FIPH animals compared to controls (*p* = 0.041 and *p* = 0.025 respectively), whilst cell density in the DG was unaffected by FIPH (*p* = 0.314 and *p* = 0.860 respectively) (Figure 2A–E). These interneuron changes in the CA1 region persisted into adulthood, with a reduced number of GAD67+ and PV+ cells per mm^2^ in FIPH animals compared to controls at P69 (*p* = 0.015 and *p* = 0.008 respectively) (Figure 2I,K). In line with the findings at P15, no abnormalities in GAD67+ or PV+ cell densities were observed in the dentate gyrus of adult FIPH rats (*p* = 0.257 and *p* = 0.287 respectively) (Figure 2J,L). We did not observe any significant changes the number SST+ cells per mm^2^ in both the CA1 and DG of FIPH rats compared to controls at P15 (*p* = 0.549 and *p* = 0.344 respectively) (Figure 2F–H). As VIP+ cells were hardly/not present in the hippocampal areas, we did not assess this subtype of interneurons in this region.

To investigate gross alterations in cortical development, DAPI+ and CTIP2+ cell counts were performed. Rats subjected to FIPH showed a significant increase in DAPI+ cells per mm^2^ throughout the cortex at P15 (*p* = 0.023), while we did not observe any changes in CTIP2+ cell density in FIPH animals (*p* = 0.845) (Figure 2M,N). To further explore which cells contribute to the observed increase in cortical cell density, a NeuN/GAD67 staining was carried out. Aside from the reported increase in GAD67+ cell density at P15, we observed a rise in NeuN+GAD67- cells per mm^2^ in FIPH animals, implying an excess of excitatory cortical neurons as well (*p* = 0.001) (Figure 2O,P). These data, in conjunction with the unchanged CTIP2+ cell density, indicate that the increased neuronal numbers are predominantly observed in layers I-IV of the rat cortex. Analysis of cortical development at P69 revealed normalization of cortical cell density in adult FIPH animals to control levels (*p* = 0.093) (Figure 2Q).

### 2.2. Intranasal MSC and IGF1 Therapy Restore Subtype-Specific Interneuron Deficits in the Cortex and Hippocampus after Postnatal Hypoxia-Ischemia and Systemic Inflammation in Mice

**Figure 3:**
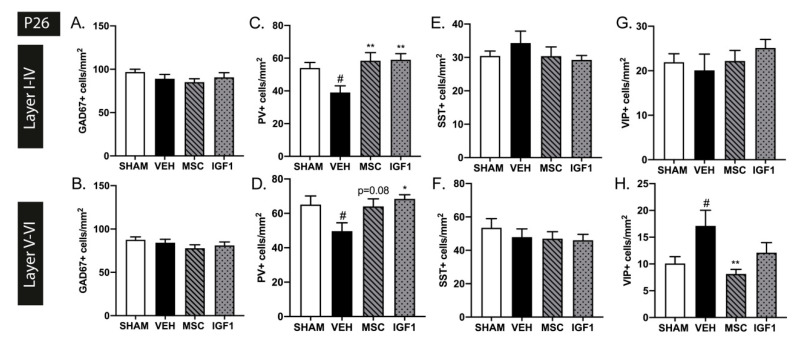
Intranasal mesenchymal stem cells (MSC) or insulin-like growth factor (IGF1) therapy reduces subtype-specific cortical interneuron deficits caused by postnatal hypoxia/ischemia and systemic inflammation in neonatal mice. (**A**,**B**) Injury induction did not lead to changes in GAD67+ cell density in the upper (**A**) or lower (**B**) cortical layers in our validated EoP mouse model at P26 (SHAM *n* = 12, VEH *n* = 13, MSC *n* = 10, IGF1 *n* = 11). (**C**,**D**) A reduction in PV+ interneuron density was observed in vehicle-treated hypoxia-ischemia and lipopolysaccharide (HI+LPS) animals (*n* = 13) compared to sham-controls (*n* = 11) in both the upper (**C**) and lower (**D**) layers of the cortex at P26. Intranasal MSC (*n* = 10) or IGF1 (*n* = 10) treatment restored cortical PV+ cell density up to sham-control levels (a trend for MSC treatment-induced recovery of PV+ cells in the lower cortical layers). (**E**,**F**) The number of SST+ interneurons was not affected in the upper (**E**) nor lower (**F**) cortex after HI+LPS (SHAM *n* = 11, VEH *n* = 10, MSC *n* = 10, IGF1 *n* = 10). (**G**,**H**) Vehicle-treated EoP mice (*n* = 12) displayed an increase in VIP+ cells per mm^2^ in the lower (**H**) but not the upper (**G**) cortical regions compared to sham-controls (*n* = 12). Treatment with intranasal MSCs (*n* = 8) significantly reduced VIP+ cell density. Intranasal IGF1 administration (*n* = 9) did not significantly affect the amount of VIP+ interneurons after EoP. #: *p* < 0.05; vehicle-treated HI+LPS animals vs. sham-controls; *: *p* < 0.05; **: *p* < 0.01; MSC- or IGF1-treated HI+LPS animals vs. vehicle-treated HI+LPS animals. Nearly significant *p* values are indicated in d.

To investigate the effect of diverse types and timing of insults on interneuron development, we used a second double-hit EoP model in newborn mice combining postnatal hypoxia-ischemia and lipopolysaccharide (HI+LPS) as recently described by our group [32]. Moreover, in this model we assessed the potential of two promising therapies in the field of EoP, namely intranasal MSCs and IGF1, to repair possible EoP-induced interneuron deficits. In contrast to the FIPH rat model, in the mouse model we did not observe any changes in cortical GAD67+ cell density in both hemispheres at 3 weeks (i.e., P26) after injury induction (Layer I-IV: *p* > 0.999 and Layer V-VI *p* = 0.648) (Figure 3A,B). This timepoint was chosen as our earlier study showed EoP-induced myelin deficits at P26 [32]. Interestingly, interneuron subtype stainings revealed a significant decrease in PV+ cells per mm^2^ in both the upper (*p* = 0.031) and lower (*p* = 0.046) cortical layers at P26 (Figure 3C,D). This deficiency in PV+ interneurons throughout the cortex was potently restored following intranasal MSC (layer I-VI *p* = 0.005 and trend in layer V-VI *p* = 0.082) and IGF1 therapy (layer I-VI *p* = 0.004 and layer V-VI *p* = 0.014) (Figure 3C,D). Similar to FIPH rats, SST+ cells density was not affected in P26 mice subjected to HI+LPS compared to sham-controls and both therapies did not show any effect either (layer I-VI *p* > 0.999 and layer V-VI *p* = 0.795) (Figure 3E,F). The decrease in PV+ cells per mm^2^ in both cortical layers in HI+LPS mice was accompanied by an increase in VIP+ interneurons in the lower cortical layers (*p* = 0.045) but not in the upper layers (*p* = 0.941) (Figure 3G,H). The increased numbers of VIP+ cells were restored to sham-control level following MSC treatment (*p* = 0.007), while IGF1 treatment did not significantly restore VIP+ cell numbers (*p* = 0.491).

**Figure 4:**
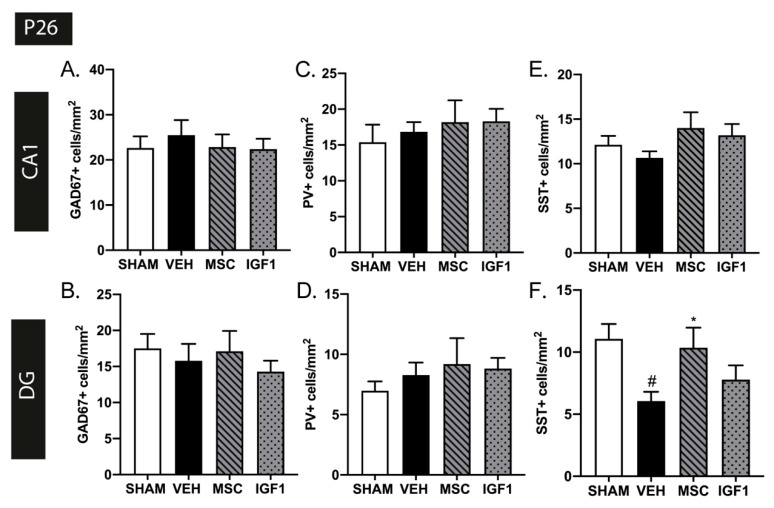
Intranasal administration of MSCs restores SST+ interneuron density deficits in the dentate gyrus. (**A**,**B**) HI+LPS induction did not lead to changes in GAD67+ cell density in the CA1 region (**A**) or dentate gyrus (**B**) of the hippocampus in vehicle-treated HI+LPS mice (SHAM *n* = 13, VEH *n* = 14, MSC *n* = 11, IGF1 *n* = 10). (**C**,**D**) HI+LPS induction did not lead to changes in PV+ cells per mm^2^ in hippocampal CA1 (**C**) or dentate gyrus (**D**) (SHAM *n* = 13, VEH *n* = 14, MSC *n* = 11, IGF1 *n* = 10). (**E**,**F**) Vehicle-treated HI+LPS animals (*n* = 10) displayed a strong decrease in SST+ interneuron density in the dentate gyrus (**F**) but not the CA1 region (**E**) compared to sham-controls (*n* = 7) at P26. Treatment with intranasal MSCs (*n* = 9) restored SST+ cell density in the dentate gyrus up to sham-control levels. Intranasal IGF1 therapy (*n* = 11) did not significantly improve SST+ interneuron density in the dentate gyrus of HI+LPS mice. #: *p* < 0.05; vehicle-treated HI+LPS animals vs. sham-controls; *: *p* < 0.05; MSC- treated HI+LPS animals vs. vehicle-treated HI+LPS animals.

In contrast to the rat FIPH model, we did not observe any hippocampal differences in GAD67+ or PV+ cell density in mice subjected to HI+LPS at P26 (CA1: *p* = 0.844 and *p* > 0.999 DG: *p* = 0.923 and *p* > 0.999 respectively) (Figure 4A–D), nor any effect of the therapies. However, a region-specific reduction in SST+ cells per mm^2^ was observed in the DG of HI+LPS mice compared to sham-controls at P26 (CA1: *p* = 0.370 DG: *p* = 0.016) (Figure 4E,F). The reduction in SST+ interneurons in the DG was restored following intranasal MSC therapy (*p* = 0.047), but not after IGF1 therapy (*p* = 0.869) (Figure 4E,F).

**Figure 5:**
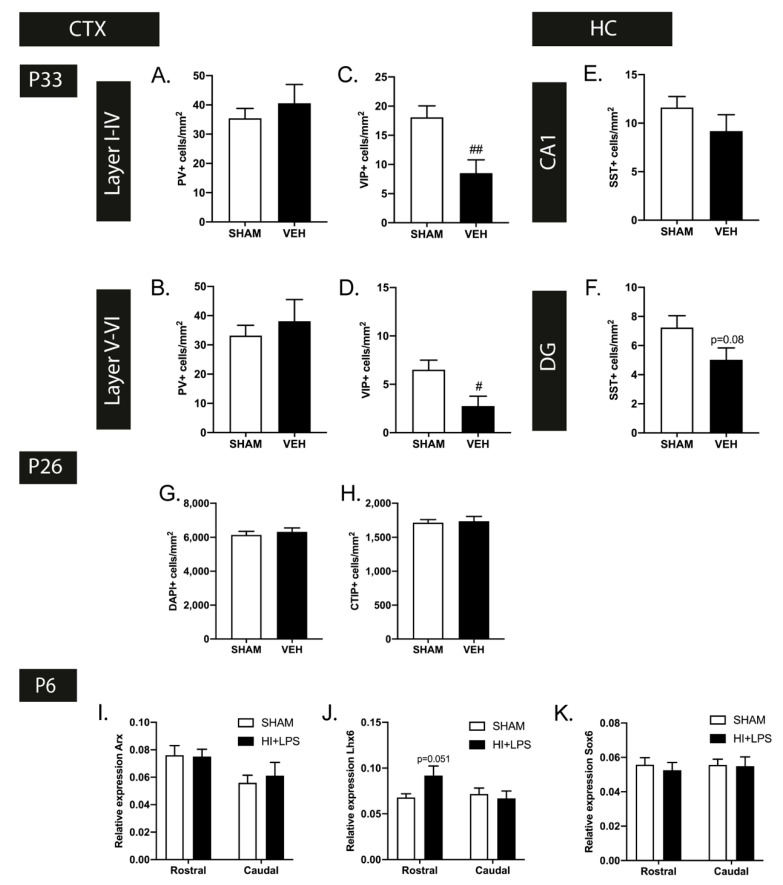
A selection of developmental disturbances in interneurons persist with age. (**A**,**B**) At P33, the observed changes in PV+ cell density were restored in both the upper (**A**) and lower (**B**) cortex (SHAM *n* = 12, VEH *n* = 9). (**C**,**D**) In contrast to the excess in VIP+ cortical interneurons at P26, a decrease in VIP+ cells per mm^2^ was observed in the upper (**K**) and lower (**L**) layers of the cortex of HI+LPS mice at P33 (SHAM *n* = 12, VEH *n* = 8). (**E**,**F**) The observed SST+ interneuron deficiency in the dentate gyrus (**F**) of HI+LPS mice showed persistence up to P33 (statistical trend), while the hippocampal CA1 region (**E**) remained unaffected (SHAM *n* = 11, VEH *n* = 8). (**G**,**H**) In contrast to the FIPH rat model, cortical cell density, measured by DAPI+ cells/mm^2^ (**G**) and CTIP+ cells/mm^2^ (**H**), was not affected at P26 by HI+LPS (SHAM *n* = 12, VEH *n* = 12). (**I**–**K**) Expression of Lhx6 (**J**) was increased (statistical trend) in the rostral cerebrum of EoP (HI+LPS) animals (*n* = 4) compared to sham-controls (*n* = 5) at 24 h after HI+LPS. Induction of EoP did not affect expression of Arx (**I**) and Sox6 (**K**). #: *p* < 0.05; ##: *p* < 0.01 HI+LPS animals vs. sham-controls; nearly significant *p* values are indicated in (**F**,**J**).

In contrast to the observations in the rat FIPH model at adult age, endogenous restoration of myelination in our mouse model, which we earlier determined at P33 [32], was not accompanied by complete resolution of interneuron deficits. Though the reduced cortical PV+ cell density at P26 was restored at P33 (layers I-IV *p* = 0.458 and layers V-VI *p* = 0.520), we observed a reduction in VIP+ interneurons in layers I-VI (*p* = 0.006) and layers V-VI (*p* = 0.020) of HI+LPS mice compared to sham-controls at P33, in contrast to P26 (Figure 5A–D). In addition, the observed deficit in SST+ interneurons observed in the hippocampal DG area of HI+LPS mice at P26, tended to persist at P33 (CA1: *p* > 0.999 DG: *p* = 0.08 (Figure 5E,F).

Contrary to the rat model, we did not observe any gross changes in DAPI+ or CTIP+ cell numbers in the cortices of HI+LPS mice compared to their sham-control littermates at P26 (*p* = 0.557 and *p* = 0.807 respectively) (Figure 5G,H), indicating that cortical density as such was not affected in the HI+LPS mouse model.

To gain more insight in the underlying pathophysiological mechanisms of interneuron deficits in EoP, we measured the expression of Arx, Lhx6, and Sox6, transcription factors reported to play a role in interneuron migration, laminar allocation and maturation [21,24], in our mouse model of EoP. At P6, i.e., 24 h after HI+LPS, we observed a borderline significant increase in Lhx6 expression in the rostral (*p* = 0.051), but not the caudal section (*p* = 0.656), of the mouse brain, while Arx and Sox6 expression was not affected (rostral Arx: *p* = 0.915 Sox6: *p* = 0.609 caudal Arx: *p* = 0.633 Sox6: *p* = 0.905) (Figure 5I–K).

### 2.3. Treatment with MSCs or IGF1 Partly Restores Sociability in EoP Mice

**Figure 6:**
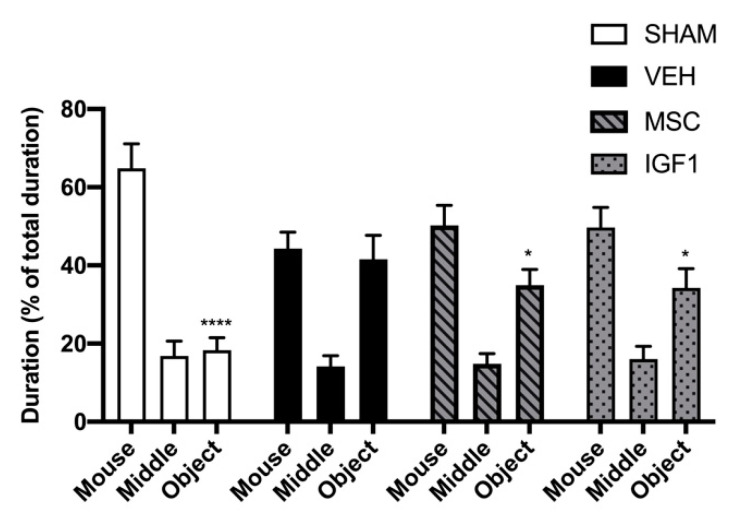
Intranasal treatment with MSCs or IGF1 partially restores sociability in an EoP mouse model. In sharp contrast to sham-operated controls, vehicle-treated EoP mice did not display a preference to spent time in the room containing the novel mouse compared to the novel object. Intranasal MSC or IGF1 partially restored sociability measured using the three chamber test (SHAM *n* = 6, VEH *n* = 7, MSC *n* = 8, IGF1 *n* = 10). *: *p* < 0.05; ****: *p* < 0.0001 time spent in room with object vs. time spent in room with mouse per experimental group.

The three-chamber task was used to quantify sociability in our EoP mouse model at P26. A deficit in social interaction is a defining feature and early marker of autism-spectrum disorders, a neurodevelopmental disorder with a higher prevalence in the (extreme) preterm infant versus the general population and often associated with interneuron maldevelopment [17,52,53,54,55,56,57]. Sham-control mice showed a strong preference for the room containing the restrained unknown mouse compared to the chamber containing the object (Figure 6, *p* < 0.0001). Interestingly, vehicle-treated HI+LPS mice failed to show a preference for either the chamber containing the restrained mouse or the chamber with the object (*p* > 0.999) (Figure 6). In other words, when comparing the % of total time spent in the chamber containing the mouse or object between HI+LPS animals and sham-controls, we observed a significant decrease in time spent with the mouse (*p* = 0.013) and significant increase in time spent with the object (*p* = 0.004), indicating reduced sociability after EoP (Figure 6). However, MSC- and IGF1-treated HI+LPS mice displayed a significant preference for the chamber containing the mouse compared to the object (*p* = 0.030 and *p* = 0.045 respectively) (Figure 6). These data imply that HI+LPS in newborn mice leads to a deficit in sociability at P26, which is partly improved by intranasal MSC or IGF1 therapy.

## 3. Discussion

In the present study, we assessed the distribution and density of interneurons in the cortex and hippocampus in two validated double-hit rodent models of EoP. We show that experimental EoP leads to distinct, model-specific patterns of disturbances in interneuron density and cortical development. Though some of the observed interneuron abnormalities were transient and restored with increasing age, a selection of developmental disturbances in interneurons was shown to persist to ages analogous to puberty or adulthood. Moreover, we demonstrated that EoP leads to a deficit in social behavior in mice, a known hallmark of both interneuron maldevelopment and preterm birth [17,55,58]. Importantly, we are the first to report the effects of two treatment strategies for EoP, i.e., intranasal application of MSCs and IGF1, on restoration of disturbances in interneuron development after EoP: both therapies showed beneficial effects on the majority of anatomical interneuron deficits and partially recovered impaired social behavior, thereby illustrating their promising potential to aid interneuron development in the preterm brain.

Using GABA as their principal neurotransmitter, interneurons are a heterogeneous population of neurons with distinct anatomical, electrophysiological and molecular features in the over 20 different reported subtypes [49]. Present in a range of brain regions, such as the cortex (~20–30% of all cortical cells) and the hippocampus (~10–15% of the neuronal population), interneurons serve as the principal source of inhibition [51,59]. Hippocampal and cortical interneurons are locally projecting cells that have been implicated in a range of processes including postnatal neuronal circuit maturation, synchronization of cortical rhythms and maintenance of the excitatory/inhibitory balance [16,48,60,61]. The role of interneurons in (postnatal) development of cortical connectivity and their association with neurodevelopmental disorders prevalent in the preterm population has led to a growing interest in interneuron abnormalities in EoP.

Consistent with previous studies that showed susceptibility of interneurons to perinatal insults in animal models and post-mortem human tissue, we observed differences in interneuron density following induction of EoP in both our models [19,20,21,23,24,25,62]. However, the previous published studies generally use single-hit (fetal/postnatal inflammation or hypoxia) models, failing to reflect the multifactorial etiology of EoP [19,23,24,25,26,27]. Furthermore, it is good to note that different studies have used different markers of subpopulations of interneurons. In our rats, the combination of fetal inflammation and postnatal hypoxia was associated with a transient increase in total cortical interneuron density. Subtype-specific stainings revealed a transient reduction in PV+ interneurons, restricted to the upper cortical layers. These changes were accompanied by an increase in cell density throughout the cortex with, aside from the observed increase in inhibitory interneurons, a transient excess of excitatory neurons (NeuN+-GAD67- cells). Moreover, we observed a reduction in hippocampal interneurons, specifically PV+ cells, in the CA1 regions of the hippocampus, persisting into adulthood. In contrast, postnatal hypoxia/ischemia and systemic inflammation at P5 in newborn mice led to a reduction of PV+ interneurons in all cortical layers, accompanied by an excess in VIP+ interneurons in the lower cortex. Though the PV+ interneuron population recovered with time, VIP+ cortical interneuron density remained abnormal at the later stage. Unlike the rat model, total cortical cell density was unaffected by EoP induction in the mouse model. In the hippocampus, a persistent reduction of SST+ interneurons was observed in the dentate gyrus of EoP mice.

Multiple explanations could account for the differences in interneuron deficits between the two EoP models used in this study and to other preclinical models. A multitude of peri- and postnatal events are believed to contribute to EoP [5,6,7,63]. Diverse patterns of interneuron injury could be the result of differences in timing and the nature of these insults, affecting distinct developmental trajectories. These diverse patterns of injury provide additional insight in the possible mechanisms and timing underlying abnormal interneuron development. First of all, the differential timing of preterm birth-related insults might have affected the generation of specific subpopulations of interneurons, as development of interneuron subpopulations is strictly regulated in time [16]. In this study, interneuron abnormalities were assessed in two distinct EoP models, a rat model incorporating fetal inflammation on E18/19 and postnatal hypoxia on P4, and a mouse model with postnatal hypoxia/ischemia and systemic inflammation on P5. In rodents, the majority of SST+ interneurons are generated during the first half of the neurogenic period in the medial ganglionic eminence (MGE), peaking around E14, whereas PV+ interneurons are produced constantly throughout neurogenesis [16]. This could explain that in experimental models of EoP, often PV+ but not SST+ interneurons are affected [19]. In line with this suggestion, we observed cortical PV+ deficits in both EoP models. However, generation of SST+ and PV+ interneurons destined for the hippocampus have been reported to peak simultaneously, making it unlikely that temporal differences in interneurogenesis account for the model-specific deficits in the hippocampus [51]. Apart from affecting interneurogenesis, preterm birth-related insults could interfere with other developmental interneuron processes, such as tangential migration, laminar allocation, programmed cell death, and maturation of interneurons. Interneurons reportedly migrate towards the cortex between E14.5 and the first postnatal days in rodents, with laminar allocation of cells continuing up to P7 [16]. Therefore, the observed deficits in PV+ interneurons in our FIPH model, limited to the upper cortical layers, might indicate disturbances in tangential migration or impaired laminar allocation after interneurons arrive at the cortical plate after inflammatory hits at E18/19. Impaired migration of interneurons has been reported in other EoP models [21]. Though interneuron progenitors express GAD67 during migration and allocation, it is only after reaching their final location in the hippocampus or cortex that interneurons mature, change morphologically and start expressing their distinctive subtype markers [64,65]. Thus, the observed increase in GAD67+ cells throughout the cortex of FIPH rats, in absence of an excess of a specific interneuron subtype could possibly reflect a maturational arrest of cortical interneurons. The net absence of GAD67+ abnormalities in our mouse model might be explained by the overshoot of VIP-expressing interneurons, masking the maturation arrest of PV+ cells. Additional evidence for interneuron maturational arrest of interneurons can be found in other preclinical studies [19,23]. In the first postnatal weeks, similar to excitatory neurons, a surplus of interneurons is eliminated via inactivity-dependent programmed cell death, leading to circuit refinement [16]. This process could be affected in our FIPH rat model, with an excess of excitatory neurons and GAD67+ cells in the cortex of injured animals. Interestingly, as the neurogenic peak of the caudal ganglionic eminence (CGE) takes place after that of its medial counterpart, loss of cortical PV+ cells in our mouse model, could possibly induce a compensatory reduction in programmed cell death of CGE-derived cells leading to the observed excessive amounts of VIP+ cells [66]. As the expression of subtype markers in our rat and mouse model was assessed at different ages (P15 vs. P26 respectively) it is possible that the excess of GAD67+ in FIPH is comprised of (immature) CGE-derived cells. Alternatively, the excess of GAD67+ cells in FIPH rats and VIP+ interneurons in EoP mice could be the result of a compensatory proliferative response after suppression of interneuron development or extensive cell loss. Tibrewal et al. [24] demonstrated increased proliferation of interneurons after preterm birth in rabbits. Moreover, Denaxa et al. [67] reported a compensatory increase in the number of interneurons derived from the caudal ganglionic eminence after loss of MGE interneurons. An explorative assessment of the expression of a small selection of transcription factors (Arx, Lhx6, and Sox6), previously shown to regulate interneuron migration and/or maturation, showed a borderline significant increase of Lhx6 expression in the rostral cerebrum of our EoP mice, at 24 h after induction of injury [24,68]. Lhx6 has been shown to regulate migration, laminar sorting and subtype marker expression of MGE-derived interneurons [68]. Lacaille et al. [21] reported similar findings, implying that this increase in Lhx6 reflects a secondary compensatory increase following an initial Lhx6 deficiency. Interestingly, in line with the findings in our EoP mouse model, total interneuron numbers remain unchanged in Lhx6 knock-out animals, as CGE-derived interneurons, such as VIP+ cells, compensate for the loss of MGE-derived cells, e.g., PV+ or SST+ interneurons [67,68]. Additional research is needed to determine if the increase in Lhx6 expression is indeed preceded by a decline in mRNA levels. Additionally, future studies should assess the full spectrum of over 20 reported transcription factors that play a role in healthy interneuron development to further elucidate the mechanisms underlying interneuron deficits in EoP [59,69]. It is important to note that variations in animal species or region of interest could play a role in the observed disparities between models, as the expression of cortical interneuron markers has been reported to differ between species and cortical regions [70]. Moreover, as mentioned previously, the age at which interneuron deficits have been assessed might play a role in the observed differences. Naturally, it is likely that the observed interneuron abnormalities are the result of a combination of these processes. Further research is warranted to elucidate the exact pathophysiology of interneuron deficits.

Similar to our previous observations in both EoP models regarding transient impairments in the white matter, the majority of interneuron deficits were also restored with age [32,50]. This could imply that in rodent models of EoP interneuron and gross cortical development is delayed rather than irreversibly damaged. Similar observations in interneuron deficits were made in other experimental models and might, to some degree, be explained by the higher regenerative capacity of the rodent central nervous system compared to humans [19,71,72]. In contrast to most cortical observations (except for the persistent cortical VIP+ interneuron aberrations in EoP mice), in both our models hippocampal interneuron abnormalities did persist with age, suggesting a different underlying pathophysiology between regional interneurons. Despite considerable endogenous restoration of interneuron density with progressing age, it is important to note that this does not necessarily equate to proper functioning. Stolp et al. [19] showed that though the absolute number of PV+ cells recovered at P40 in a model of inflammation-induced diffuse WMI, the number of interneurons with perineuronal nets, a morphological marker of functional maturity, was significantly lowered. Similarly, Thion et al. [71] reported impaired synaptic inhibition by interneurons, while cell density had recovered. Thus, even after normalization of interneuron numbers, inhibitory control of the cortex could be permanently affected after EoP. Moreover, even if interneuron numbers and functioning is transiently disturbed, cerebral circuit development could be irreversibly affected [73]. For example, a reduced number of interneurons has been associated with reduced input to subcortical nuclei, leading to persistent volumetric deficits [14]. In line with this hypothesis, we observed volumetric gray matter deficits in our EoP mouse model [32]. Additional studies that include functional outcome parameters, such as morphology, including the formation of perineural nets and electrophysiology of cortical networks, are needed to determine the long-term effects of preterm birth-related insults on interneuron functionality.

To our knowledge, we are the first to report on the regenerative potential of intranasal MSC and IGF1 therapy to restore aberrant interneuron development in a model of EoP. Aside from our earlier data showing an important role of intranasal MSCs and/or the boosting potential of IGF1 on white matter development [32], here both therapies were shown to restore the majority of interneuron abnormalities after EoP induction in mice. These intranasal therapies therefore seem to target a broad spectrum of processes affected by preterm birth-related insults, thereby aiding in proper development of both the white and gray matter after EoP. Moreover, EoP mice treated with MSCs or IGF1 showed significantly more social interaction, though, after both therapies, their sociability was not completely restored up to sham-control levels. In line with the findings in our mouse model of EoP, we previously demonstrated impaired social behavior in FIPH rats [50]. Thus, the observed deficits in interneuron development in cortex and hippocampus in our EoP models, and the beneficial effects of the therapies on these deficits could partially explain the partial improvement in social behavior. However, multiple brain regions have been implicated to play a role in social functioning, including the prefrontal cortex, basal ganglia, and amygdala and hippocampus [17,74]. Aside from focal lesions, brain injury in other (distant) areas has been shown to impact anatomical structure and functioning of the brain regions involved in social functioning [75]. Furthermore, prior to restoration by regenerative therapies, short-lived aberrant interneuron development, locally or in other connecting brain regions, could possibly still affect long-term (social) functioning. In accordance with this hypothesis, we observed persistent volumetric deficits of the hippocampus, despite early restoration of myelination after intranasal MSC therapy [32]. Moreover, though multiple studies have established an association between social dysfunction and interneuron deficits, we cannot assume 1:1 causality as other underlying mechanisms of EoP might also affect brain regions implicated in social behavior [14]. Though impaired social functioning is a key hallmark of neuropsychiatric disorders that are associated with preterm birth, including ASD, additional studies are needed to determine if our EoP mice exhibit an ASD-like phenotype, including the assessment of other typical ASD behaviors, such as repetitive grooming.

Based on this study, intranasal MSC therapy shows slight superiority in restoration of interneuron abnormalities compared to intranasal IGF1 therapy. This observation might be explained by the plethora of beneficial (growth) factors secreted by MSCs in the EoP microenvironment compared to one beneficial factor [32]. In our previous study examining the potential of MSCs to restore WMI after EoP, we showed that in vitro exposure of MSCs to the EoP milieu induced distinct secretome changes in MSCs [32]. We observed an upregulation of growth factors associated with interneuron migration and/or maturation, such as hepatocyte growth factor (HGF), nerve growth factor (NGF), and glial cell line-derived neurotrophic factor (GDNF) [76,77,78,79]. Thus, it is possible that this plethora of MSC-secreted factors underlie the observed regenerative effect of MSCs after EoP. In addition, MSC and IGF1 treatment possibly affect different processes of interneuron development and thereby outcome, due to differences in the timing of administration and/or mechanism of action of the treatments. Though effective, it is currently unclear through which mechanism(s) of action these therapies boost interneuron development. It is possible that the MSCs’ secretome or IGF1 directly positively affect interneurons; for example, neuregulins, HGF, GDNF and IGF1 have been implicated in interneuron development [40,77,78,79,80,81]. Moreover, an indirect effect of the treatment, through dampening of neuroinflammation for instance could also contribute to proper interneuron development. For example, astrogliosis and subsequent secretion of BMPs has been shown to halt development of the SST+ interneuron subtype [82]. More insight into the working mechanisms of MSC and IGF1 therapy could be obtained using primary interneuron cultures. Interestingly, restoration of interneuron development could even underlie the earlier observed repair of white matter deficits, as interneurons have been reported to regulate oligodendrocyte maturation by emittance of pro-differentiation cues through transient synaptic input and secreted factors [83,84]. Due to the multifactorial etiology of EoP and distinct clinical course in each patient, it is unlikely that a single animal model represents the entirety of EoP-associated injury. The substantial differences in the pattern of interneuron deficits that were observed in our models and other studies could reflect the range of interneuron deficits that can result from preterm birth. Therefore, the efficacy of MSC or IGF1 therapy to restore interneuron abnormalities should best be confirmed in additional models of EoP.

In summary, induction of EoP led to model-specific alterations in interneuron development, indicating that the vulnerability of interneuron subtypes to preterm birth-related insults is dependent on the specific developmental time-window. Moreover, both intranasal MSCs and IGF1 are promising therapeutic agents to restore atypical interneuron development. This study provides a step forward in understanding the full spectrum of pathophysiological mechanisms underlying EoP and shows the potential of two regenerative therapies to reduce interneuron maldevelopment in the (extreme) preterm brain.

## 4. Materials and Methods

### 4.1. Animal Models of Encephalopathy of Prematurity

All animal procedures were carried out according to the Dutch and European guidelines (Directive 86/609, ETS 123, Annex II) and were approved by the Experimental Animal Committee Utrecht (Utrecht University, Utrecht, The Netherlands) and the Central Authority for Scientific Procedures on Animals (the Hague, The Netherlands) (project identification code: AVD115002016751, date of approval: 1 December 2016). Animals were kept under standard housing conditions with food and water available ad libitum in a temperature-controlled environment. Both sexes were included in all experiments and randomly assigned to each experimental group.

#### 4.1.1. Rat Model

EoP was induced as described previously [50]. In short, timed-pregnant Wistar rats (Envigo, Horst, The Netherlands) received an intraperitoneal (i.p.) injection of 100 µg/kg lipopolysaccharide (LPS) (Sigma, L2880, Saint Louis, MO, USA), dissolved in sterile 0.9% NaCl, on embryonic day (E) 18 and 19. After birth, postnatal day (P) 4 rat pups were exposed to hypoxia (8% O_2_) during 140 min in a temperature-controlled hypoxic chamber. Control dams received 0.9% NaCl i.p. injections on E18 and E19 (1 mL/kg). On P4, control offspring was placed in a normoxic temperature-controlled environment for 140 min. Rats were euthanized by an i.p. overdose of pentobarbital (250 mg/kg) at P15 (i.e., 11 days after EoP induction) and P69 (i.e., 65 days after EoP induction). These time points were based on the previously observed kinetics of dWMI in this model with transient myelination deficits present at P15 and endogenously restored at P69 [50].

#### 4.1.2. Mouse Model

EoP was induced in C57BL/6j mouse pups using a combination of hypoxia-ischemia (HI) and systemic inflammation, as described recently by our group [32], which is an adaptation of the model originally described by from Shen, Plane, and Deng [85]. At P5 HI was induced by permanent unilateral occlusion of the right common carotid artery under isoflurane anesthesia (5–10 min; 5% induction, 3% maintenance with flow O_2_: air 1:1). After a 75-min recovery period, pups were subjected to hypoxia (6% O_2_) during 35 min under temperature-controlled conditions (35.8–36.0 °C). Subsequently, animals received an i.p. injection of 1mg/kg LPS (List Biological Laboratories, Campbell, CA, USA), dissolved in sterile 0.9% NaCl. Sham-control littermates underwent isoflurane anesthesia and incision only, without carotid artery occlusion, nor hypoxia nor LPS injection. Mice were euthanized by an i.p. overdose of pentobarbital (250 mg/kg) at P26 (i.e., 21 days after EoP induction) or P33 (i.e., 28 days after EoP induction). Similar to the rat model, we based these time points on our previous study, with evident white matter injury at P26 and endogenous restoration of the observed deficits at P33 [32].

### 4.2. Treatments in the Mouse EoP Model

#### 4.2.1. Mesenchymal Stem Cells

Purchased GIBCO^®^ mouse (C57BL/6) bone marrow-derived MSCs (Invitrogen, S1502-100; Carlsbad, California, USA) were cultured according to the manufacturer’s instructions. For all treatments, cells were passaged once (from P2 to P3) prior to administration. Three days after EoP induction (P8), mice received 2 dosages of 2 µL hyaluronidase (12.5 U/µL in total, Sigma-Aldrich, St. Louis, MO, USA) dissolved in H_2_O in each nostril (total of 8 µL). Hyaluronidase improves the permeability of the nasal mucosa and is therefore routinely used to facilitate cell delivery [86]. Thirty minutes after hyaluronidase administration, 0.5 × 10^6^ MSCs in dPBS (Thermo Fisher, 14190-169, Waltham, MA, USA) were administered intranasally to the mouse pups, in 2 dosages of 2 µL in each nostril (total of 8 µL). dPBS administration was used as a vehicle treatment. Dose and timing of MSC treatment were based on our earlier study [32].

#### 4.2.2. Insulin-Like Growth Factor 1

Directly following EoP induction, P5 mice received intranasal IGF1 treatment during six consecutive days (i.e., on P5-P10). Mouse pups received a daily dosage of 25 µg recombinant human IGF1 (PeproTech, 100-11, Cranbury, NJ, USA) dissolved in sterile 0.9% NaCl, in 2 nose drops of 2 µL in each nostril (total of 8 µL). Vehicle-treated animals received similar volumes of intranasal 0.9% NaCl.

### 4.3. Arx, Lhx6, and Sox6 Expression in EoP Mice

Sham-control and HI+LPS (untreated) brains were collected at P6, and cerebrum was divided in rostral and caudal sections. Brain sections were snap-frozen in liquid nitrogen and stored at −80 °C until further processing. Sections were crushed using a mortar and pestle chilled on liquid nitrogen. RNA was isolated using the RNeasy Mini kit (Qiagen, 74104, Hilden, Germany) with on-column DNase digestion with the RNase-free DNase set (Qiagen, 79254, Hilden, Germany). RNA quantity and quality were assessed by spectrophotometry (NanoDrop 2000, Thermo-scientific, Waltham, MA, USA) at 260 mm and OD 260/280 ratio. Subsequently, cDNA transcription was performed using iScript Reverse Transcription Supermix for RT-qPCR (Bio-Rad, 1708840, Hercules, CA, USA). Real-time qPCR was carried out with SYBR Select Master Mix (Thermo Fisher Scientific, 4472908, Waltham, MA, USA) on the QuantStudio 3 Real-Time PCR System (Thermo Fisher Scientific, A28136, Waltham, MA, USA). Primers sequences can be found in Table 1. Mean expression of GAPDH and β-actin were used for data normalization.

### 4.4. Three Chamber Social Test in EoP Mice

The three-chamber social test was used to evaluate same-sex sociability. The task, originally described by Nadler et al. [87], was carried out with some adaptations. A rectangular, clear polycarbonate box with three chambers, (all 42.5 cm W × 22.2 cm H; center chamber: 17.8 cm L; side chambers: 19.1 cm L) and dividing walls with retractable doorways, allowing access into each chamber, was used to assess social behavior. Same-sex, unknown (non-litter mates), sham-control mice were used as stimulus mice and were restricted in inverted wire-metal cages that were placed in one of the side-chambers, such that experimental animals had to enter the chamber to initiate social interaction. Testing consisted of two consecutive 10-min stages: (1) habituation in all chambers with empty wire metal cages and (2) the social novelty phase; with the wire cages containing an object or the stimulus sham-control mouse. The location of the stimulus mouse was randomized between mice to avoid side preferences. The experimental set-up was cleaned using a soap solution between all animals to eliminate olfactory stimuli. Investigators were blinded for experimental groups during assessment and analyses. The number of entries and time spent in each of the compartments (center chamber or side chambers with either mouse or object) was measured and calculated as a percentage of total time or chamber entries respectively.

### 4.5. Immunohistochemistry

Following an overdose of pentobarbital, rats or mice were transcardially perfused using PBS followed by 4% PFA in PBS. Brains were post-fixed during 24 h in 4% PFA, followed by a dehydration ethanol series and paraffin embedment. Coronal sections (8 µm) were cut at the level of the hippocampus (−1.8 mm from bregma) in mice and rats, and at the level of the lateral ventricles (bregma) in rats. These brain areas were selected as we observed deficits in the white matter here previously in the respective models [32,50]. For immunofluorescence stainings, sections were deparaffinized using xylene and rehydrated in decreasing concentrations of ethanol. For antigen retrieval, sections were heated to 95 °C in sodium citrate buffer (0.01M, pH 6). After cooling down and three PBS/0.05% Tween20 (PBS-T) washes, non-specific binding was blocked using 5% normal donkey serum in PBS-T. Subsequently, sections were incubated overnight with primary antibodies diluted in PBS (see Table 2 for details and dilutions). The next day, sections were washed three times in PBS and incubated with alexa fluor 594 and 488 conjugated secondary antibodies (Life Technologies, Carlsbad, CA, USA; 1:500) for 75 min at room temperature. Sections were counterstained using DAPI (1:5000) and embedded in FluorSave (Merck Millipore, 345789, Burlington, MA, USA).

### 4.6. Microscopy and Image Analysis

Investigators were blinded for experimental conditions during image acquisition and analyses. Sections were imaged using a Cell Observer microscope with an AxioCam MRm camera (Zeiss, Oberkochen, Germany). Images of both hemispheres were taken using the 10x objective. In each hemisphere, cortical layers were imaged in two series of two (mouse; four images in total at −1.8 mm from bregma) or three (rat; six images in total at bregma) adjacent micrographs (covering the full height of the cortex, layer I-VI) at a fixed distance from the cingulum. In addition, 10× images of the dentate gyrus and CA1 region were obtained in the hippocampal sections. For each animal, the upper (I-IV) and lower (V-VI) cortical layers were delineated based on CTIP2+ staining, a marker primarily expressed by neurons residing in layers V-VI, using AxioVision Rel. 4.8.2 software (Zeiss, Oberkochen, Germany). Subsequently, cells with a clear DAPI+ nucleus and the marker of interest (i.e., either GAD67, PV, SST or VIP) were counted manually using ZEN 2012 software (Zeiss, Oberkochen, Germany) in both the cortex and hippocampus. Areas containing excessively high background staining, non-specific staining, or substantial artefacts were excluded. For the NeuN/CTIP2 staining the total amount of CTIP2+, NeuN+, and DAPI+ cells was counted automatically using the analyze particles plugin for ImageJ v1.47. Cell counts were corrected for the total area per region of interest (upper or lower cortical layers, CA1 or DG) and averaged per animal. Despite unilateral carotid artery occlusion in the mouse model; we did not observe any differences in cortical interneuron density between hemispheres (data not shown). Thus, from here onwards, the reported cortical interneuron density represents the average of both hemispheres in both rats and mice. Due to unilateral hippocampal area loss in our mouse model [32], hippocampal interneurons were counted in the hemisphere contralateral to carotid artery occlusion.

### 4.7. Statistics

Data is presented as mean ± standard error of the mean (SEM). All statistical analyses were carried out using GraphPad Prism 8.3 software. Unpaired t-tests, or in the event of unequal variances non-parametric Mann–Whitney tests, were used for the comparison of two groups. One-way ANOVA with Bonferroni post-hoc tests, or a non-parametric Kruskal–Wallis test with Dunn’s post hoc correction in case of unequal variances, was carried out for comparison of multiple groups. *p*-values < 0.05 were considered statistically significant. Specific sample sizes are mentioned in the figure captions.

## Figures and Tables

**Table 1:** Overview of primers used in the study.

Symbol	Forward Primer Sequence	Reverse Primer Sequence
Arx	GCACCACGTTCACCAGTTAC	TCTGTCAGGTCCAGCCTCAT
Lhx6	CGTTGAGGAGAAGGTGCTTTGC	GCTTGGGCTGACTGTCCTGTTC
Sox6	ATCTCTCATCCCGGCCTAAGAC	TCCCCAGGCTTCCTCCAATG
GAPDH	TGAAGCAGGCATCTGAGGG	CGAAGGTGGAAGAGTGGGAG
β-actin	AGAGGGAAATCGTGCGTGAC	CAATAGTGATGACCTGGCCGT

**Table 2:** Overview of primary antibodies used in the study.

Antigen	Species (Host)	Company, Product Code	Dilution
Anti-GAD67	Mouse	Sigma, MAB5406	1:200
Anti-PV	Mouse Rabbit	Sigma, P3088 Novusbio, NB120-11427	1:1000 1:200
Anti-SST	Mouse	Santa Cruz, SC-74556	1:50
Anti-VIP	Rabbit	ImmunoStar, 20077	1:1000
Anti-CTIP2	Rat	Abcam, ab18465	1:1000
Anti-NeuN	Mouse Rabbit	Merck Millipore, mab377 Abcam, ab177487	1:200 1:500

## Data Availability

The data presented in this study are available on request from the corresponding author.

## Abbreviations

EoP	Encephalopathy of Prematurity
MSC	Mesenchymal Stem Cell
IGF1	Insulin-like Growth Factor I
PV	Parvalbumin
SST	Somatostatin
VIP	Vasoactive Intestinal Polypeptide
GAD	Glutamate Decarboxylase
WMI	White Matter Injury
ADD	Attention Deficit Disorder
ASD	Autism Spectrum Disorder
FIPH	Fetal Inflammation and Postnatal Hypoxia
HI+LPS	Hypoxia/Ischemia and Lipopolysaccharide
DG	Dentate Gyrus
CA	Cornu Ammonis
P	Postnatal Day
i.p.	Intraperitoneal
LPS	Lipopolysaccharide
SEM	Standard Error of the Mean
HI	Hypoxia-ischemia
HGF	Hepatocyte Growth Factor
GDNF	Glial Cell Line-Derived Neurotrophic Factor
NGF	Nerve Growth Factor
MGE	Medial Ganglionic Eminence
CGE	Caudal Ganglionic Eminence
